# Altruism and anti-anthropocentrism shape individual choice intentions for pro-environmental and ethical meat credence attributes

**DOI:** 10.1371/journal.pone.0294531

**Published:** 2023-11-28

**Authors:** Sven Anders, Marina Malzoni, Henry An

**Affiliations:** Department of Resource Economics and Environmental Sociology, University of Alberta, Edmonton, Alberta, Canada; Public Library of Science, UNITED KINGDOM

## Abstract

Food consumption patterns are changing as consumers are becoming more aware and interested in sustainable and ethical food production practices. The growing disconnect between primary (livestock) agriculture and society reinforces the importance of research examining the motivations behind consumer purchase behaviors. However, evidence that links latent consumer psychometric factors and observed heterogeneity in concerns for agriculture to individual food purchase intentions remains scarce. We employ large-scale survey data and an advanced Structural Equation Modelling approach to identify and estimate the direct and indirect effects of latent fundamental values and observed consumer characteristics on individuals’ attitudes and purchase intentions for certified humane (CH), organic, and non-hormone added labeled meat products. Our findings suggest that human values, including self-transcendence and openness to change, drive farm animal welfare concerns and individuals’ choices of certified meat products. Information and engagement in social media positively affect individuals’ perceptions and concerns for farm animal welfare. Individuals guided by altruistic and anti-anthropocentric norms are more oriented towards sustainable and ethical food shopping behaviors.

## 1. Introduction

Among the many challenges facing the agricultural sector, the most preoccupying ones are the competition for resources, climate change, and the public’s growing mistrust [1]. This last issue is due to a widening gap between how some segments of modern society want the agricultural sector to operate versus how the modern industrial agricultural sector operates. Mindful consumers–such as those who read the information on food labels–still represent a niche segment but are gaining market clout and are already capable of shaping conventional agricultural practices [2]. As consumers are starting to influence how their food is produced, understanding the reasons behind their purchases has become vital to anticipating and meeting their demands.

Given the growing focus on sustainability, ethics, and social aspects of agriculture, how the agricultural sector operates is in the spotlight of agri-food sector debates. Specifically, when we look at livestock production, consumers are increasingly paying attention to attributes attesting animal welfare and the sustainability of meat production [3]. While some individuals may decide to stop eating meat altogether, other consumers can compensate for the emotional distress by taking comfort in knowing that the animal was humanely treated before and during slaughtering [4]. In this study, we examine Canadian consumer meat choice behavior as livestock agriculture occupies an important place in Canadian society and culture [5]. Moreover, the beef sector has been plagued by a series of disruptive events (e.g., BSE in 2003, E. coli outbreak in 2012), which have altered societal perceptions of agriculture and especially animal production [6]. In 2014, Canada’s second largest grocery retailer Sobeys and fast-food hamburger chain A&W introduced “Certified Humane” (CH) labeled meat products. Analyzing how Canadian consumers first reacted to this innovation provides insight into understanding the evolution of value-based market niches in Canada and offers guidance to other markets currently undergoing similar market transitions.

Previous literature has sought to analyze the drivers of consumer decision-making towards certified meat, with a growing attention to the role of values, norms, and other psychometric variables that comprise the mental processes influencing behavior [7, 8]. The concept of personal norms, first introduced by Schwartz in 1977 [9], guided one of the first insights into self-interest, altruism, and biosphere values as drivers of consumer environmental concerns—which have only recently received greater attention in the literature. However, psychometric variables are difficult to quantify, and it is not always clear how they influence consumer intentions. This issue poses a problem in moving the literature in consumer behavior forward [10].

The objective of this paper is to analyze the role fundamental values play in shaping consumer purchase intentions regarding CH, organic, and non-hormones added labeled meat in Canada. We use a two-stage econometric approach to estimate how different guiding life principles and human values can explain farm animal welfare (FAW) concerns, and act as drivers of consumer purchase intentions towards certified meat. Our empirical analysis employs a Multiple Indicators Multiple Causes (MIMIC) Structural Equation Model (SEM) that enables measuring relationships between multiple latent variables and multiple observable variables [11], while avoiding the risk of measurement bias associated with the translation of ordinal and Likert-scale based latent constructs into observed covariates within single equation models [12]. The MIMIC approach thus allows us to consider the influence of multiple latent variables simultaneously, whereas previous studies on consumer behavior and FAW [13, 14] could only consider a single latent construct in their SEM. Our analysis of how the psychometric variables altruism and anti-anthropocentrism jointly influence consumer choice behavior for sustainable meat options that address consumer FAW concerns hold implications for food marketing and policy and contributes to our conceptual understanding of how basic human values affect meat choice behaviors.

## 2. Literature review

Food consumption patterns and shopping behavior are changing in response to concerns about environmental resources, sustainability, and animal welfare [2, 15]. Accordingly, food labels (e.g., organic, CH, etc.) are an effective tool providing detailed information about a specific good, mitigating consumer concerns, and driving purchase decisions, especially when it comes to pro-environmental and ethical attributes. As a result, some scholars have examined how consumers respond to pro-environmental and ethical food attributes [16, 17].

There are some well-known drivers of consumer behavior towards green labels in the literature: people who identify as female, vegetarians, and pet-owners are more likely to choose labels that highlight ethical and sustainable practices [18–20]. In addition, people with more children and higher income lean towards green labels [21]. Consumer trust and a transparent production process can also positively impact individual perceptions and attitudes [22]. Moreover, knowledge, education, eating habits, religion, and environmental concerns are all factors that influence sustainable and ethical food purchases [23–26].

Despite this extensive knowledge, there is a growing interest in understanding the psychometric drivers and fundamental values behind preferences for food (meat) labels. Psychometric factors seek to measure the mental process influencing behaviors and can be applied to estimate the effects of latent variables, which are unobservable but inferred from other observed metrics [27]. Meat demand differs from the demand for other types of food as it comes from a living creature raised and killed for human consumption. Consequently, meat consumption incorporates a moral decision for some consumers [4, 28]. Thus, consumer ethics [29], personal norms [30], moral concerns, and attitudes [31, 32] are the major constructs influencing shopping behaviors towards pro-environmental and FAW meat credence attributes.

The difficulty with measuring such psychometric drivers arises from the necessity to infer their influence through related observed variables [27, 33]. SEM methods have been extensively used in consumer research for its ability to consider both direct and indirect effect pathways between latent and observed variables affecting behavioral outcomes [34] and to gain deeper insight into consumer behavior towards pro-environmental and ethical goods [13, 16] local foods [35], and the role of altruism behind the purchase of green packaging [36]. However, research that employs SEM methods to estimate how human values drive sustainable food choices or those focusing on the roles psychometric factors, such as altruism and anti-anthropocentrism, play in consumer choice decisions within a SEM framework are lacking [13].

The relationship between psychometric variables and food choices is not a new concept in the literature [37, 38]. Schwartz in 1992 developed one of the most well-known scales for measuring individual’s life-guiding principles, which is still widely applied to food behavioral analysis. Schwartz [39] elaborated on four meta-values- values that combine the results of multiple scientific studies -explaining different consumer profiles: conservation (security, conformity, and tradition) versus openness to change (stimulation, self-direction, and hedonism) and self-enhancement (hedonism, achievement, and power) versus self-transcendence (benevolence and universalism). It is still a debate whether hedonism is more related to openness to change or to self-enhancement, but empirical evidence shows that it may be best attributed to openness to change [40–42]. As a result, several scholars have linked the heterogeneity among meat-eating habits with these human values using Schwartz’s approach [7, 40, 41]. S1 Table provides detailed information about the values of Schwartz’s theory.

Several studies find positive relationships between self-transcendence and openness to change as drivers of consumer behavior towards organic and FAW attributes [7, 40, 42]. On the other hand, conservation [40] and self-enhancement, which is related to egoism, are minor concerns for individuals who care about FAW and natural resources preservation [7, 43]. The self-transcendence component of the Schwartz scale has been widely used to measure altruism, with universalism being also closely related to pro-environmental attitudes [44]. Altruism is motivated by acting in accordance with one’s moral values and concern for the welfare of others [45]. Universalism is the broader form of altruism, while benevolence is related to the welfare of close others [46]. We measured altruism by the self-transcendence component of Schwartz scale since it was found to make individuals focus on the interests of others and the environment [39] and are usually related to pro-environmental attitudes [47]. On the other hand, anthropocentrism, in a narrower sense, refers to the belief that humans are the primary holders of moral standing [48]. According to previous scholars, an anti-anthropocentric profile has been shown to be a driver of organic meat purchases [49].

The literature has examined both altruism and anthropocentrism in studies measuring attitudes towards the environment [48], consumer pro-environmental and ethical purchase intentions [50] and FAW [51]. Hence, understanding the role that altruism and anti-anthropocentrism play is important to assessing the drivers of CH meat choices amidst growing competition with plant-based meat or protein alternatives. Due to the development of novel behavioral economic approaches [52], the role of human values and personal norms in food purchase decisions is receiving increasing attention in the literature. Nevertheless, empirical evidence on how these multiple subjective factors relate to FAW concerns and subsequent food choice behaviors is lacking. This is particularly true for how self-identity and other latent constructs relate to human values.

## 3. Conceptual framework

Understanding how human values, altruism and anti-anthropocentrism, relate and influence on consumers’ behaviors is gaining importance to understand their role on pro-environmental attitudes. When investigating behaviors, researchers often refer to attitudes and norms by employing the Theory of Planned Behavior (TPB). TPB postulates that cognitive, attitudinal, personal, and social factors can predict observed behaviors. However, previous studies have also found human values to be important factors driving both concerns and (meat) choice behaviors [7, 40]. According to Schwartz [53], values are central components of our personality and motivators of behaviors and attitudes. Several different scholars have developed their own instruments to measure value [39, 54, 55]. We measured human values following Schwartz’s Theory of Basic Human Values (TBHV). Schwartz’s framework is built on the concept of “universal values” and has previously been applied in several consumer studies towards FAW [40, 56]. This literature confirms that Schwartz’s values reinforce the linkage between attitudes, values, and behavior, making the TBHV the best-validated framework for assessing human values.

**Fig 1:**
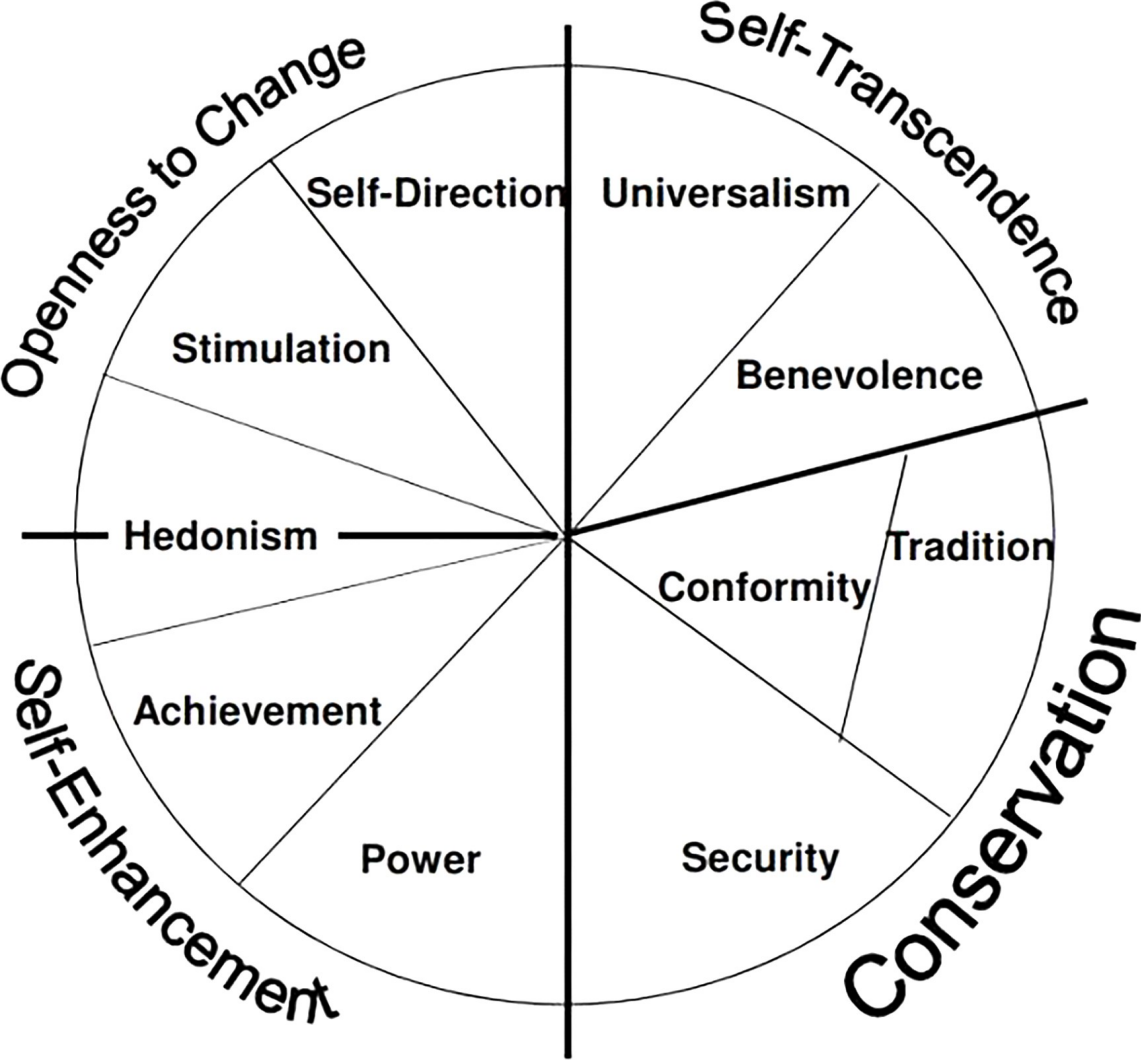
Key meta-values according to the Theory of Basic Human Values (adapted from Schwartz, 1992).

Schwartz’s framework allows us to develop our econometric and statistical analysis by measuring core meta-values, such as self-transcendence and self-enhancement. Our choice of the Schwartz approach also rests on its solid place in the literature and on our belief that it measures human values in a greater detail as it concerns the basic values that people in all cultures recognize. According to Schwartz [39, 53] human values are solid beliefs represented in a circular structure based on four core meta-values: self-enhancement, self-transcendence, openness to change, and conservation (Fig 1). The position of these indicators within the circle provides information about the similarities and differences among them. For example, proximity along the circular structure indicates similar meanings, while distance signifies conflict or contrast [8].

Previous studies have applied the TBHV to understand how these human values are related to organic and FAW attributes preferences [40]. These studies found openness to change and self-transcendence as drivers of consumer concerns for pro-environmental and ethical food attributes, whereas self-enhancement is negatively related to green labels purchases. At the first stage, our research aims to contribute to this literature by measuring how Schwartz’s meta-values are related to FAW concern, while estimating the effects of self-identity and other personal norms.

Following Schwartz’s scale, we use the meta-value self-transcendence to measure altruism. We also adopt the New Ecological Paradigm (NEP) scale [57], which was developed to assess the different levels of environmental concern that significantly shape attitudes and has been widely applied to examining consumer behavior [58]. Specifically, we use selected items of the NEP scale to account for the belief that humans and animals have the same right to exist (anti-anthropocentrism). That differs from ecocentrism, which is related to the perspective that places importance on the ecosystem [59]. We applied the concept of anti-anthropocentrism following previous scholars, who found a higher concern about animal welfare to be significantly related to the anti-anthropocentrism dimension of the NEP Scale [60]. Although there are discussions surrounding the validity of the NEP scale [33], it remains the most frequently used measure of environmental concern and that is why we decided to apply this approach in our study. While altruism was measured following the TBHV, anti-anthropocentrism was measured according to the NEP scale items. Finally, we also hypothesized that altruism is a driver of anti-anthropocentrism since altruism was found to be positively related to pro-environmental attitudes by previous scholars [34].

The difficulty with measuring psychometric variables arises from the necessity to infer their influence through related observed variables [27, 35]. SEM methods have been extensively used in consumer research for their ability to consider both direct and indirect effect pathways between latent and observed variables affecting behavioral outcomes [36], to gain deeper insight into consumer behavior towards pro-environmental and ethical goods [13, 16] local foods [61], and the role of altruism behind the purchase of green packaging [62]. However, research that employs SEM methods to estimate how human values drive sustainable food choices or those focusing on the roles psychometric factors, such as altruism and anti-anthropocentrism, play in consumer choice decisions within a SEM framework are lacking [13].

A common feature of existing applications of SEM that infer the effects of latent psychometric constructs using one or more observed variables is the use of Likert-scale data based on survey questionnaires. These SEMs typically treat these Likert responses as continuous variables in single equations models of effect pathways, which can result in measurement bias when modeling discrete dependent variables that require non-linear estimation procedures [12]. To overcome this problem, the MIMIC model [63] allows researchers to relate latent variables (reflective measures) to their indicators (formative measures) and observed respondent characteristics and uses a choice model that estimates the effect of each latent and observed variable on an individual’s decision [64]. The MIMIC approach not only reduces measurement bias, but also allows researchers to more accurately assess the performance of formative constructs; in other words, validate the process by which latent variables mediate the effects of observed respondent characteristics on outcomes of interest [65, 66]. In the context of this paper, specifying the latent variables of altruism and anti-anthropocentrism as mediators, we can estimate both the direct and indirect impact (via the latent factors) of respondent characteristics and survey responses labeled meat choice decisions. This method thus provides a causal structure to our understanding of the drivers of ethical and environmentally motivated food behaviors, rather than measuring the effects of each observed variable individually while controlling for the effects of latent variables.

Advanced SEM modelling using MIMIC is a key component to assessing how fundamental values affect human sustainability behaviors. To that end, we implement a two-tiered empirical analysis. Stage 1 tests Schwartz’s meta-values as drivers of citizens’ perceptions and FAW concerns, while controlling for socio-demographics, knowledge, experience, and personal norms. We hypothesize that altruism and anti-anthropocentrism are the main values driving concerns about FAW. Stage 2 then analyzes how select fundamental human values from Schwartz’s scale shape consumer purchase behaviors towards certified meat products using the MIMIC SEM approach.

The econometric specification of our MIMIC SEM approach mitigates this risk by allowing multiple latent variables to mediate the effects of multiple observed variables, thus improving the consistency and reliability of the pathways effect estimates in explaining Canadian’s frequency of choosing CH, organic and non-hormone labeled meat products. For example, liberalism may directly increase the likelihood by which an individual chooses CH meat. However, political views may also be related to certain latent variables, such as altruism, which can act as an indirect pathway influencing green label purchases. MIMIC SEM allows us to understand and account for these multiple dependencies between explanatory constructs, which is particularly important for analyzing what motivates consumers to buy CH meat.

**Fig 2:**
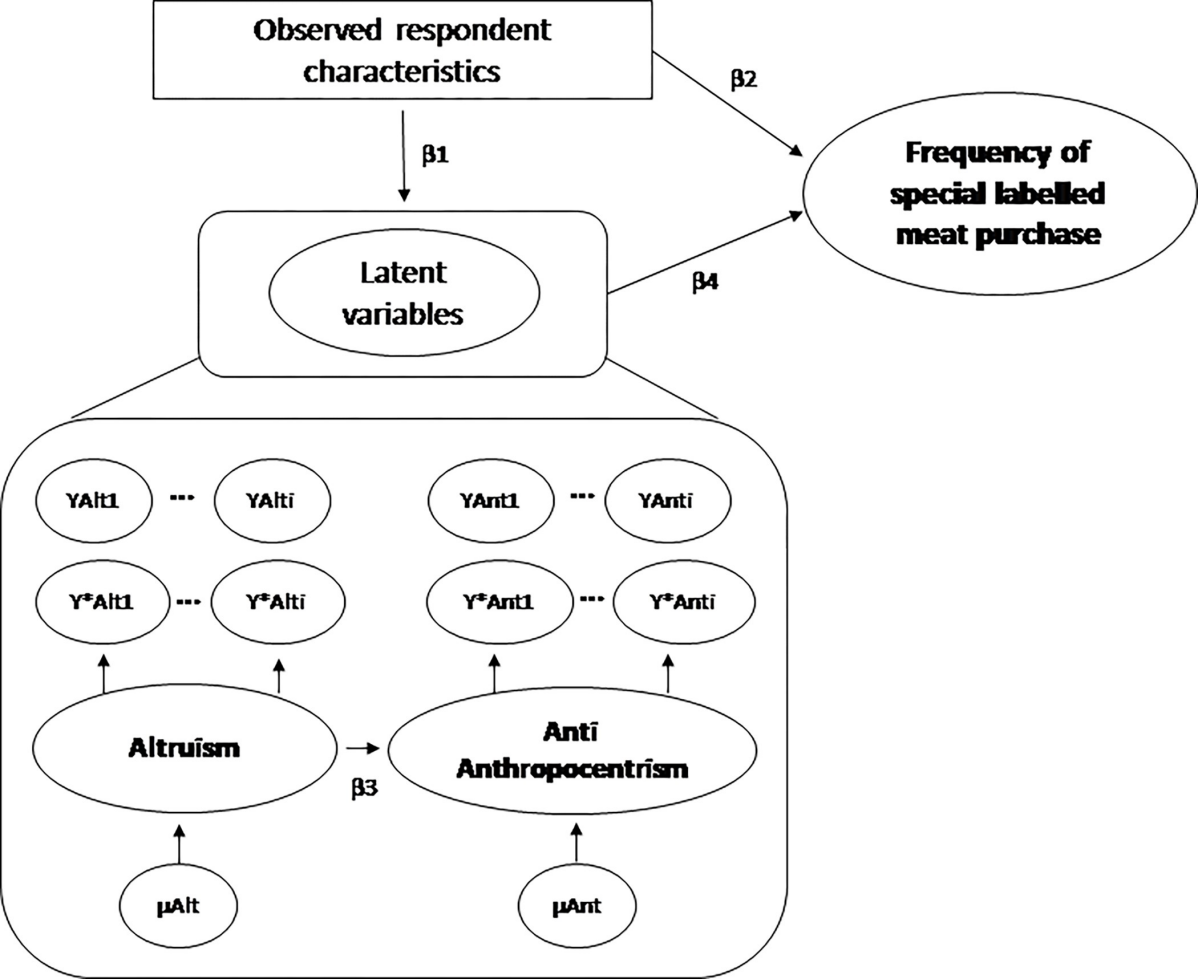
Path diagram estimating observed and latent variables effects on intentions to purchase certified humane meat.

Following [21, 67], attitudes were measured via shopping intentions, knowledge, experience, and FAW concerns. In addition, we controlled for other lifestyle factors, such as spiritualism, ethics, political views, and social media engagement. Fig 2 presents the structural model to be estimated and the path diagram of the effects of observed and latent variables on respondents’ purchase intentions. Arrows represent causal relationships between variables, rectangles indicate observed variables, and ovals correspond to latent constructs.

We hypothesize that a combination of observed and latent variables is driving heterogeneous purchase decisions. Following an extensive literature, we hypothesize that certain observed characteristics are positively related to the frequency of special labeled meat purchases, such as gender [18], number of children [21], income [68] and politics and ethics [69]. Besides the well-known effects of observed variables, we hypothesize that altruism and anti-anthropocentrism are also driving certified meat choices. This is in line with previous scholars who found altruism and anti-anthropocentrism to be positively related to FAW concerns [51, 60]. Following [34], we expect altruism to be a driver of anti-anthropocentrism since altruism was found to drive pro-environmental attitudes. Finally, we aim to estimate the relationship between observed respondent characteristics and latent variables, such as the relationship between altruism and anti-anthropocentrism with gender [70].

## 4. Empirical approach

The survey data was collected in 2016 by the research company Ipsos-Reid. The questionnaire was designed to collect information about the motivations driving food purchase behavior for different meat labels. The study received human research ethics (IRB) approval at the University of Alberta, #Pro00062848. Written consent was obtained before participants entered the study. No personal identifying information was elicited as part of the study and the analysis was conducted on anonymous data. The survey elicited responses from a sample of 1,602 Canadian consumers invited from a panel of 200,000 households across the ten Canadian provinces—The survey considered only English speakers across the ten Canadian provinces. Sample quotas for gender, age, income, and education assured representativeness of the English-speaking Canadian population based on 2016 census data from Statistics Canada [71].

Table 1 provides sample descriptive statistics. The survey questionnaire is described in more detail in S2 Table. The 40-minute questionnaire consisted of sections containing questions related to food shopping patterns, individuals’ level of concern for FAW, patterns of and reasons for purchasing CH labeled meat, knowledge about livestock agriculture, personal values, and social media usage. The survey sections focusing on FAW engagement followed [5, 72]. At the beginning of the survey, respondents were asked how frequently they purchased different types of meat (organic, hormone-free, CH) on a 3-point Likert scale. Our measure of respondents’ attitudes and knowledge is similar to [73]. Agricultural knowledge was measured in two ways: (1) subjectively, by asking the respondent to compare his knowledge against that of his social circle; and (2) objectively, by asking each respondent a set of randomized questions related to modern agriculture. We also asked respondents how certain they were about their answers, which allowed us later to exclude probable correct answers due to random luck that are not due to higher agricultural knowledge.

**Table 1:** Descriptive statistics of the survey respondent sample.

	Average	S.D.	%	Min	Max	Description
*Male*	0.49		49.16%	0.00	1.00	1 if male
*Age*	47.20	15.26		18.00	86.00	Age in years
*Vegetarian*	0.11		11.16%	0.00	1.00	1 if vegetarian
*Pet Owner*	0.58		58.39%	0.00	1.00	1 if pet owner
*Income*	7.68	5.11		0.50	25.00	Income in ’00,000 CAD
*FAW Concern*	3.80	0.96		1.00	5.00	5-point Likert scale
*Liberal*	0.37		36.90%	0.00	1.00	1 if liberal
*Conservative*	0.24		23.81%	0.00	1.00	1 if conservative
*Subjective Ethics*	3.50	0.88		1.00	5.00	5-point Likert scale
*Social Media Use*	0.42		41.5%	0.00	1.00	1 if use social media
*University*	0.24		24.13%	0.00	1.00	1 if attended University
*Subjective Agricultural Knowledge*	0.23		22.64%	0.00	1.00	1 if believe know more about agriculture
*Altruism*	4.27	0.84		1.00	5.00	5-point Likert scale
*Anti-anthropocentrism*	3.50	1.06		1.00	5.00	5-point Likert scale
*Religious*	0.35		34.77%	0.00	1.00	1 if religious
*Purchase frequency (CH)*	1.77	0.80		1.00	3.00	3-point Likert scale
*Purchase frequency (Organic)*	1.80	0.74		1.00	3.00	3-point Likert scale
*Purchase frequency (Hormone-free)*	2.02	0.83		1.00	3.00	3-point Likert scale
*Lived on a farm*	0.16		16.26%	0.00	1.00	1 if lives on a farm
*British Columbia*	0.17		17.29%	0.00	1.00	1 if lives in British Columbia
*Prairies*	0.22		21.81%	0.00	1.00	1 if lives in Prairies
*Ontario*	0.49		48.52%	0.00	1.00	1 if lives in Ontario
*Atlantic*	0.08		8.06%	0.00	1.00	1 if lives in Atlantic

We developed 5-point Likert scale questions to measure respondents’ level of concern for FAW, as well as their perceptions across Schwartz’s guiding life principles (1992). Likert scale questions can capture whether respondents’ purchases are driven primarily by concern for FAW or by a desire for a higher quality of life (S3 Table). In this regard, self-enhancement measures the respondent’s belief that humans have the right to modify the environment and their desire for having power and being wealthy. Conservation is correlated with greater respect for older individuals, while self-transcendence is associated with justice. Openness to change is represented by the desire to have a varied and eventful life. Items measuring altruism are composed of Schwartz’s meta-value representing self-transcendence, especially the aim for justice, peace, and equality. By employing items from the NEP scale, we measured anti-anthropocentrism by eliciting individual perceptions of whether humans are meant to rule over nature [57]. The items forming each latent scale and their response formats are listed in Table 2.

**Table 2:** Description of scales used to measure latent variables.

Variable	Question	Likert-Scale
Altruism	Please think about how important each statement is as a guiding principle in your life	Not at all important - Not very important—Neutral—Somewhat important—Very important
ALT1	A world at peace, free of war and conflict	
ALT2	Equality, equal opportunity for all human beings	
ALT3	Social justice, correcting injustice and caring for the weak	
Anti-anthropocentrism	Please indicate whether you agree or disagree with the following statements	Strongly disagree—Agree
AA1	Humans have the right to modify the natural environment to suit their needs	
AA2	Humans were meant to rule over the rest of nature	
AA3	Plants and animals have as much right as humans to exist	
Purchase frequency	How often have you purchased meat products with any of the claims listed below?	
PF1	Certified Humane	Never—Rarely—Regularly
PF2	Organic	
PF3	Hormone free	

Ordered logistic regressions have been widely employed within food economics and marketing research. In the first stage, the dependent variable is the respondents’ indicated level of concern for FAW. Therefore, the model specification is:

$$y_{i}=\beta_{1}*Sociodemographics+\beta_{2}*Vegetarian+\beta_{3}*Trustin\;farmers+\beta_{4}*Social\;media\;engagement+\beta_{5}*Ethics+\beta_{6}*Religion+\beta_{7}*Liberalism+\beta_{8}*Norms\;and\;Human\;values+\varepsilon_{i},$$ (1)

where *y*_*i*_ is the FAW concern to be represented by the vector of observed variables (socio-demographics, vegetarianism, trust in farmers, social media engagement, ethics, religion, liberalism, and human values), and *ε*_*i*_ is the error term. βs are parameters to be estimated.

The dependent variable *y*_*i*_ can be segregated into thresholds, as each respondent has a level of FAW concern. The following process underlines the mapping of the latent attitude, with *i* referring to each respondent and *j* representing one of the five responses for the FAW concern model:

$$y_{i}=1(not\;at\;all\;concerned),\;if\;y_{i}\leq 1$$

$$y_{i}=2(not\;very\;concerned),\;if\;1<y_{i}\leq \mu_{1}$$

$$y_{i}=3(neutral),\;if\;\mu_{1}<y_{i}\leq \mu_{2}$$

$$y_{i}=4(somewhat\;concerned),\;if\;\mu_{2}<y_{i}\leq \mu_{3}$$

$$y_{i}=5(very\;concerned),\;if\;\mu_{3}\leq y_{i},$$ (2)

where the parameters *μ*_*i*_ are the externally imposed endpoints of the observable categories. Assuming that the errors are independently and identically distributed, the probabilities models can be represented as the following:

$$Prob(y_{i}=1)=\alpha (-\beta x_{i})$$

$$Prob(y_{i}=2)=\alpha (\mu_{1}-\beta x_{i})-\alpha (-\beta x_{i})$$

$$Prob(y_{i}=3)=\alpha (\mu_{2}-\beta x_{i})-\alpha (\mu_{1}-\beta x_{i})$$

$$Prob(y_{i}=4)=\alpha (\mu_{3}-\beta x_{i})-\alpha (\mu_{2}-\beta x_{i})$$

$$Prob(y_{i}=5)=1-\alpha (\mu_{3}-\beta x_{i})$$ (3)

$$L(\beta ,\mu )=\sum_{j}\sum_{i}I[y_{i}=j]log(\alpha (\mu_{j}-\beta x_{i})-\alpha (\mu_{j}-1-\beta x_{i}),$$ (4)

where *β*_*i*_ and *μ*_*i*_ are chosen to maximize the log-likelihood equation presented in Eq 4. Existing studies have applied a similar analysis to understand consumer interests [74], and different determinants of consumption behavior towards organic [75] and FAW [76] labeled products. We use this approach to understand how human values shape perceptions regarding FAW while controlling other observed variables. We estimate the multiple factors that drive concern for FAW in Canada by estimating an ordered logistic regression using the *ologit* procedure in the statistical software package Stata [77].

### Estimating direct and indirect drivers of certified humane meat purchases

The SEM of respondent purchase intentions towards CH labeled meat is modeled using a vector of observed variables (e.g., socio-demographics) and three latent variables represented by altruism, anti-anthropocentrism, and subjective purchase frequency. We implement this SEM using the MIMIC approach discussed above in the software package Mplus Version 8 [78]. Brief descriptions of the latent groups are provided in Table 2.

The intention to purchase organic, hormone-free, and CH labeled meat is assumed to be driven by an unobserved function, Z*, which is modeled using the following ordered logit model:

$$Z^{*}=\beta X+\gamma L+\varepsilon$$

$$Z^{*}=\left\{ \begin{array}{l} 1\;if\;Z^{*}\leq k_{1}\\ 2\;if\;k_{1}<Z^{*}\leq k_{2}\\ 3\;if\;k_{2}<Z^{*}, \end{array}\right.$$ (5)

where *X* is a vector of observed variables, *L* is a vector of latent characteristics, β and *γ* are parameters to be estimated, and ε is the error term. More precisely, *L* is composed of the latent variables, altruism, anti-anthropocentrism, and purchase intention. Two thresholds, *k*_1_ and *k*_2_, are estimated to predict the observed categorical response.

Eqs 6 and 7 represent the measurement component of the MIMIC model and estimates the relationship between the *i*^*th*^ indicator to the *j*^*th*^ latent variable as shown in Table 2. The MIMIC model assumes there is an underlying response $y_{ij}^{*}$ explaining individual *i*’s response to the c-category indicator question, *Y*_*ij*_, while estimating *c*−1 thresholds or *π*. This relationship is demonstrated below:

$$y_{ij}^{*}=\theta_{i}L_{j}+v_{ij},\;\theta_{i}=1$$ (6)

$$Y_{ij}=\left\{ \begin{array}{l} 1\;if\;{Y_{ij}}^{*}\leq \pi_{ij,1}\\ 2\;if\;\pi_{ij1}<{Y_{ij}}^{*}\leq \pi_{ij,2}\\ \;.\\ \;.\\ \;.\\ c\;if\;\pi_{ij,c-1}<{Y_{ij}}^{*} \end{array}\right.$$ (7)

The equations above are estimated using probit models, where *π*_*i*_ is a matrix of coefficients and *v*_*ij*_ represents the matrix of error terms. The second component of the MIMIC model is the structural model based on the relationship of each latent variable (*L*_*j*_) to the observed constructs (*X*) as shown below:

$$L_{j}=\partial_{j}X+u_{j},$$ (8)

where ∂_*j*_ and *u*_*j*_ are parameters and residuals, respectively. This system of equations is computed in *Mplus* 7.0.

## 5. Results and discussion

### Ordered logit regression of FAW concerns

The marginal effects of the ordered logistic regression results examining the factors driving individuals’ concerns for FAW are provided in Table 3. Estimated coefficients and goodness-of-fit test statistics are provided in S4 Table. We find that anthropocentrism is negatively related to FAW concern, whereas individuals with a self-transcendence profile are more likely to care about the well-being of farm animals. Consistent with [8], we find that a positive attitude toward change and having exciting life experiences can increase the likelihood of advocating for ethically produced food by 2.87 percentage points. Additionally, we find that individuals who perceive themselves as ethical and politically liberal are more likely to care about FAW in accordance with previous results [32, 69]. According to our results, women are more concerned about FAW, possibly because they are more inclined to buy green label products as found by previous study [18]. Moreover, the positive effect of having children also aligns with other scholars [21]. In line with previous work [79, 80], we find minor statistical significance of socioeconomic variables compared with personal norms and attitudes.

**Table 3:** Ordered logit marginal effects results for farm animal welfare concerns.

	1	2	3	4	5
	Not at all concerned	Not very concerned	Neutral	Somewhat concerned	Very concerned
**Socio-Demographics**
Female	-0.00954***	-0.0392***	-0.113***	0.0227**	0.139***
	(0.0021)	(0.0061)	(0.0150)	(0.0090)	(0.0184)
Age	-0.00014***	-0.00056***	-0.00165***	0.00039**	0.00196***
	(4.97e-05)	(0.0002)	(0.0005)	(0.0002)	(0.0006)
Number of children	-0.00247*	-0.0102*	-0.0301*	0.00707*	0.0357*
	(0.0013)	(0.0053)	(0.0154)	(0.0043)	(0.0183)
Income	9.74e-09	4.04e-08	1.19e-07	-2.79e-08	-1.41e-07
	(1.34e-08)	(5.52e-08)	(1.62e-07)	(3.91e-08)	(1.93e-07)
Vegetarian	-0.00306*	-0.0128*	-0.0400*	0.00252	0.0534*
	(0.0016)	(0.0066)	(0.0217)	(0.0043)	(0.0325)
**Knowledge and Experience**
Trust in farmers	2.67e-05	0.000111	0.000326	-7.65e-05	-0.00039
	(0.0007)	(0.0028)	(0.0084)	(0.0020)	(0.0100)
Education	0.000496	0.00206	0.00606	-0.00142	-0.00719
	(0.0004)	(0.0015)	(0.0044)	(0.0011)	(0.0053)
Seek FAW information	-0.0120***	-0.0505***	-0.163***	-0.0548***	0.280***
	(0.00243)	(0.00596)	(0.0143)	(0.0194)	(0.0307)
Engage in social media	-0.00288**	-0.0120**	-0.0355**	0.00737*	0.0429**
	(0.0013)	(0.0050)	(0.0147)	(0.0038)	(0.0181)
**Norms and Human Values**
Ethical	-0.00451***	-0.0187***	-0.0551***	0.0129***	0.0653***
	(0.0011)	(0.0034)	(0.0093)	(0.0048)	(0.0107)
Religious	-0.00110**	-0.00458**	-0.0135**	0.00317*	0.0160**
	(0.0005)	(0.0020)	(0.0058)	(0.0017)	(0.0068)
Liberal	-0.00170***	-0.00706***	-0.0208***	0.00489**	0.0247***
	(0.0006)	(0.0022)	(0.0063)	(0.0022)	(0.0074)
Belief that humans rule over nature	0.00366***	0.0152***	0.0447***	-0.0105***	-0.0530***
	(0.0009)	(0.0027)	(0.0073)	(0.0039)	(0.0084)
Being influential is important	-0.00078	-0.00325	-0.00956	0.00225	0.0113
	(0.00071)	(0.0029)	(0.0085)	(0.0021)	(0.0101)
Justice is important	-0.00315***	-0.0131***	-0.0385***	0.00903**	0.0456***
	(0.0010)	(0.0035)	(0.0100)	(0.0038)	(0.0117)
Respect for elders is important	-0.00102	-0.00425	-0.0125	0.00294	0.0149
	(0.0008)	(0.0034)	(0.0100)	(0.0025)	(0.0119)
Having an exciting life is important	-0.00198**	-0.00820***	-0.0242***	0.00568**	0.0287***
	(0.0008)	(0.0031)	(0.0090)	(0.0028)	(0.0107)
Being wealthy is important	0.00051	0.00213	0.00626	-0.00147	-0.00743
	(0.0006)	(0.0027)	(0.0079)	(0.0019)	(0.0093)
Observations	1,329	1,329	1,329	1,329	1,329

*, **, *** significant at 10%, 5%, 1% level, respectively.

The influence of household and individual eating habits is included in the analysis. Although vegetarians are not meat consumers, they can still be meat shoppers for the household. In line with previous studies [20], our results show that vegetarians are more likely to be concerned about the living standards of farm animals. Furthermore, the level of trust in farmers is negatively related to FAW concern but is not statistically significant. In line with previous findings [25], we also find that social media is an important driver of FAW concern.

### Structural equation model results

As a first step in the statistical process, we list the measurement model results in Eq 8 (Table 4), which examine the relationship between latent variables and their predictors as described in Table 2. The model fit statistics, specifically the Root Mean Square Error of Approximation (RSMEA = 0.034, ≤ 0.05) and the Comparative Fit Index (CFI = 0.954, ≥ 0.9) suggest that our empirical model fits the data well [81]. Additionally, loadings have highly significant coefficients on their corresponding latent group, which implies validity and reliability of the model. The ordinal alpha scores [82] range from 0.66 for anti-anthropocentrism to 0.79 for altruism, implying an acceptable internal consistency of each scale.

**Table 4:** Results of the measurement model relating latent variables to their indicators.

Latent Variable	Indicator	Coefficient	S. E.
*Altruism* *(α = 0*.*79)*	Alt1	1	0
	Alt2	1.130***	0.04
	Alt3	1.006***	0.035
*Anti-Anthropocentrism* *(α = 0*.*66)*	AA1	1	0
	AA2	1.098***	0.055
	AA3	0.886***	0.041
*Purchase Frequency* *(α = 0*.*78)*	PF1	1	0
	PF2	0.886***	0.031
	PF3	1.080***	0.038
**Residual Variance**			
*Altruism*		0.502***	0.028
*Anti-Anthropocentrism*		0.399***	0.026
*Purchase Frequency*		0.584***	0.029

*, **, *** significant at 10%, 5%, 1% level, respectively.

Table 5 presents the effects of individual’s observed traits on the latent constructs. We find both altruism and anti-anthropocentrism influence consumer behavior toward certified meat. Hence, personal characteristics that are closely associated with altruism and anti-anthropocentrism may also indirectly explain purchase behavior involving pro-environmental and ethical meat. Our findings reveal that being vegetarian is directly related to a higher purchase intention of CH, organic and non-hormone added meat. However, we also find vegetarians to be statistically less altruistic. Although previous literature states that vegetarianism is associated with altruism and biocentrism [83], our findings suggest that survey respondents change their eating habits mostly for their own health and safety concerns, as they are less concerned about justice, peace, and equality. One point worth repeating is that only vegetarians that are also meat buyers are included in this analysis, which may explain the differences between our results and those from other studies.

**Table 5:** Linear model estimates with latent traits as the dependent variables.

Observed Variables	Altruism		Anti-Anthropocentrism		Purchase Frequency	
Variable	Coefficient	S. E.	Coefficient	S. E.	Coefficient	S. E.
Very Likely to Examine Food Labels					1.079***	0.092
Likely to Examines Food Labels					0.574***	0.1
Purchases Meat Weekly					0.15***	0.052
Subjective Agricultural Knowledge					0.174***	0.061
Social Media Usage					0.019	0.033
Male	-0.329***	0.048	-0.224***	0.046	0.05	0.052
Age	0.012***	0.002	-0.002	0.002	-0.008***	0.002
Vegetarian	-0.2***	0.077	-0.109	0.068	0.25***	0.076
Pet Owner	0.036	0.049	0.249***	0.045	0.125**	0.053
Income	-0.002	0.005	0	0.004	0.011**	0.005
Religious/Spiritual	0.171***	0.05	-0.24***	0.044	0.225***	0.052
University	-0.173***	0.059	-0.11**	0.052	0.057	0.062
British Columbia	-0.118*	0.064	0.092	0.062	0.157**	0.067
Prairies	-0.041	0.058	-0.016	0.053	-0.027	0.062
Atlantic	-0.048	0.09	0.048	0.07	-0.041	0.094
Liberal	0.21***	0.056	0.04	0.05	0.131**	0.057
Conservative	-0.058	0.059	-0.149***	0.057	0.111*	0.066
Suburban	0.038	0.058	-0.048	0.051	0.022	0.061
Concern about FAW	0.104***	0.033	-0.007	0.03	0.086	0.037
Subjective Ethics	0.415***	0.033	0.062**	0.031	-0.083**	0.039
Lived on a Farm	0.068	0.049	-0.064	0.046	0.109**	0.051
Altruism			0.263***	0.032	0.136***	0.042
Anti-Anthropocentrism					0.165***	0.046
R^2^	0.65		0.48		0.68	

*, **, *** significant at 10%, 5%, 1% level.

We find pet-owners to be more likely to purchase CH, organic, and non-hormone added labeled meat, which the results from Table 5 suggest is partly due to their anti-anthropocentrism nature in line with others [19]. We also find that individuals who consider themselves ethical are more altruistic and anti-anthropocentric than others. In this sense, although ethical identity does not directly enhance the probability of purchasing CH labeled meat, it can hold an indirect influence due to its linkage with human values [61]. Beyond that, this analysis also examines respondents’ political orientation. We find that respondents who perceive themselves as liberal are more likely to prefer labels attesting FAW, directly and indirectly, due to altruism. On the other hand, conservative individuals are more anthropocentric, which negatively affects their intentions toward CH, hormone-free, and organic meat (Table 5).

In agreement with the literature [68], we also find that household income directly influences consumer behavior towards specially labeled meat. While the relationship between income and FAW concern is statistically insignificant, we find that a high level of income positively affects actual purchases of specially labeled meat. However, income is still only a minor contributor as a driver of shopping decisions towards sustainable and ethical meat when compared to other values and constructs, such as political orientation and knowledge. These results also provide knowledge related to the value-action gap since consumers state to have a high level of FAW concern, which is not fully translated into choice actions yet. According to [84], consumers with a high income are more likely to purchase sustainable items, so a lower income can be perceived as a barrier for consumers to act in line with their concerns and engage in sustainable purchases. In addition, while older individuals were found to be more concerned about FAW, younger respondents are the ones actually purchasing sustainable and ethical certified meat with more frequency.

Table 6 presents estimates of direct, indirect, and total variable effects. Indirect effects capture the relationship between our observed and latent variables, telling us how our observed variables influence the intention to purchase specially labeled meat mediated through latent variables (altruism and anti-anthropocentrism). The direct effect can be interpreted as each observed variable’s impact on the purchase frequency of CH, organic and non-hormone added meat, while controlling for altruism and anti-anthropocentrism. From Table 6, we observe that concerns about FAW directly and indirectly influence individuals to purchase CH, organic and hormone-free meat with the indirect component mainly due to its linkage with altruism, as shown in Table 5.

**Table 6:** Direct, indirect, and total effect estimates of all variables on individuals’ intention to purchase certified humane labeled meat.

	Indirect Effect	Direct Effect	Total Effect	
Variables	Coef.	Coef.	Coef.	S. E.
Male	-0.096***	0.050	-0.046	0.050
Age	0.002***	-0.008***	-0.006***	0.002
Vegetarian	-0.054***	0.250***	0.196***	0.076
Pet Owner	0.048***	0.125**	0.173***	0.052
Suburban	-0.001	0.022	0.021	0.061
Income	0.000	0.011**	0.01**	0.005
Religious/Spiritual	-0.009	0.225***	0.216***	0.051
University	-0.049***	0.057	0.007	0.062
British Columbia	-0.006	0.157**	0.151**	0.067
Prairies	-0.01	-0.027	-0.037	0.063
Atlantic	-0.001	-0.041	-0.042	0.094
Liberal	0.044***	0.131**	0.175***	0.057
Conservative	-0.035**	0.111*	0.076	0.066
FAW Concern	0.018**	0.086**	0.104***	0.037
Subjective Ethics	0.085***	-0.083**	0.001	0.037
Lived on a Farm	0.002	0.109**	0.111**	0.052
Subjective Agricultural Knowledge		0.174***	0.174***	0.061
Highly Examines Food Labels		1.079***	1.079***	0.092
Moderate Examines Food Labels		0.574***	0.574***	0.100
Purchases Meat Weekly		0.150***	0.150***	0.052
Social Media Usage		0.019	0.019	0.033
Altruism		0.136***	0.136***	0.042
Anti-Anthropocentrism		0.165***	0.165***	0.046

*, **, *** significant at 10%, 5%, 1% level, respectively.

In accordance with [26], our results in Table 6 also show that individuals who live on a farm and claim to have higher (subjective) farm knowledge are also more likely to purchase CH, hormone-free, and organic labeled meat. Similarly, we find that highly educated and well-informed individuals are more likely to purchase meats with these labels. Religious consumers are more oriented towards specially labeled meat, directly and indirectly, as they are more altruistic. However, individuals with higher levels of spiritualism are also found to be more anthropocentric, which should negatively affect their purchase intentions towards CH meat [23, 24]. In general, our results provide evidence that religious Canadian consumers are more likely to engage in sustainable shopping decisions, since they are more altruistic. However, there is also a negative component driving their purchase intentions, as they are also more likely to be anthropocentric.

Overall, these results provide evidence that eating habits, income, religion, political views, knowledge, and social media engagement are directly related to the purchase frequency of CH, organic, and non-hormone added labeled meat. However, we also find an indirect component mediated through the latent variables, altruism, and anti-anthropocentrism. The decision-making process towards FAW certified meat is complex, and the SEM results from this study provide evidence about how explanatory variables are related as drivers of specific meat shopping intentions.

In this regard, Table 7 reveals the indirect effects of observed variables on the purchase frequency of certified labeled meat. This table provides the effect of each observed variable on the purchase frequency due to its connection with altruism and/or anti-anthropocentrism. For example, religious individuals are more likely to purchase CH meat, as they are shown to be more altruistic. Nonetheless, they also hold a negative indirect component, as religious individuals are more likely to be anthropocentric. These results are important since it provides a detailed analysis of the pathways driving consumers to engage in mindful shopping conducts.

**Table 7:** Statistically significant indirect effect pathways for purchase frequency (PF) of certified humane labeled meat.

Path of indirect effects on PF	Coefficient	S. E.
Male → ALT → AA → PF	-0.014***	0.005
Male → ALT → PF	-0.045***	0.015
Male → AA → PF	-0.037***	0.013
Age → ALT → AA → PF	0.001***	0
Age → Alt → PF	0.002***	0.001
Vegetarian → ALT → AA → PF	-0.009**	0.004
Vegetarian → AA → PF	-0.027**	0.013
Pet Owner → AA → PF	0.041***	0.014
Religious → ALT → AA → PF	0.007**	0.003
Religious → ALT → PF	0.023**	0.01
Religious → AA → PF	-0.04***	0.013
University → ALT → AA → PF	-0.008**	0.003
University → AA → PF	-0.023**	0.011
University → AA → PF	-0.018*	0.01
Liberal → ALT → AA → PF	0.009**	0.004
Liberal → AA → PF	0.028**	0.012
Conservative → AA → PF	-0.025**	0.012
**FAW Concern → ALT → AA → PF**	0.005**	0.002
FAW Concern → ALT → PF	0.014**	0.006
Ethics → ALT → AA → PF	0.018***	0.006
Ethics → ALT → PF	0.056***	0.018
Ethics → AA → PF	0.01*	0.006

*, **, *** significant at 10%, 5%, 1% level, respectively.

## 6. Conclusions

Our main objective was to contribute to the conceptual understanding of how psychometric variables in the form of basic human values explain individuals’ FAW concerns and the role they play as latent drivers of consumer choice of certified meat products in the Canadian market. Consistent with our hypothesis and aligned with [7, 40] we find basic human values, altruism and anti-anthropocentrism influence both FAW concerns and sustainable meat choices. At the same time, consumers who read food labels and process this information are more likely to engage in pro-environmental and ethical behaviors—favouring pro-environmental and ethical attributes. Our results build on those of [7, 40] by focusing on Canadian consumers during a period in which FAW and CH programs were first introduced in retail and chain-restaurant markets, emphasising the joint effects latent values, information, and knowledge play in shaping consumer responses to sustainable products options in meat. Moreover, our application of a MIMIC SEM model fills a methodological gap in the literature by jointly estimating the pathway effects of multiple latent variables using multiple observable demographic variables as indicators to more reliably determine how values enter into food (meat) choice decisions.

Understanding consumer values holds implications for current food marketing and policy that increasingly seeks to speak to individuals’ values and norms in attempts to shift food behaviors towards greater sustainability and climate awareness. Following a large consumer literature, our results confirm the role information continues to play in shaping food choices. Food labels presenting clean, objective, and concise information that contributes to informed decision-making can strengthen the marketing of CH labeled meat, and possible other value-based labeled [16]. Moreover, we show that information goes beyond food labels, as social media engagement was a precursor of FAW concern already in 2016. Social media effects may have intensified over time, and the influence social media has on consumer food behavior remains a pressing research topic.

Finally, by using a MIMIC SEM, we were able to identify and estimate direct and indirect pathway effects of multiple latent constructs through multiple observed variables regarding purchase intentions for sustainable and FAW centered meat options. Reliable estimates of the dependencies between psychometric factors, eating habits, social media engagement, knowledge in their influence on product choice intentions provides an academic contribution. It also may be applicable to the understanding of the intrinsic motivations behind emerging shifts in consumption patterns such as the move to plant-based protein alternatives. The main contribution of our results is that they provide specific knowledge regarding how personal norms and self-identity constructs are driving Canadian shopping decisions towards specific meat label.

Nonetheless, further analysis of how values and norms, such as selfishness versus altruism, influence individual food behaviors remains an important topic of research. We invite future research in this area to consider employing the MIMIC SEM approach to minimize the risks of measurement error and resulting flawed conclusions regarding consumer attitudes and behaviors towards food system sustainability. Such knowledge would lead to more effective marketing and policy strategies that are better aligned with consumer interests.

## Supporting information

S1 Table Motivational values of Schwartz theory of basic values. (TIF)

S2 Table Structure and objectives of the survey questionnaire. (TIF)

S3 Table Description of survey variables. (TIF)

S4 Table Parameter estimation of the FAW concern ordered logit model. (TIF)
