# Supplementary figures and images for: Altruism and anti-anthropocentrism shape individual choice intentions for pro-environmental and ethical meat credence attributes
Source: PLoS One. 2023 Nov 28;18(11):e0294531. doi: 10.1371/journal.pone.0294531 (PMC10684090; doi:10.1371/journal.pone.0294531)

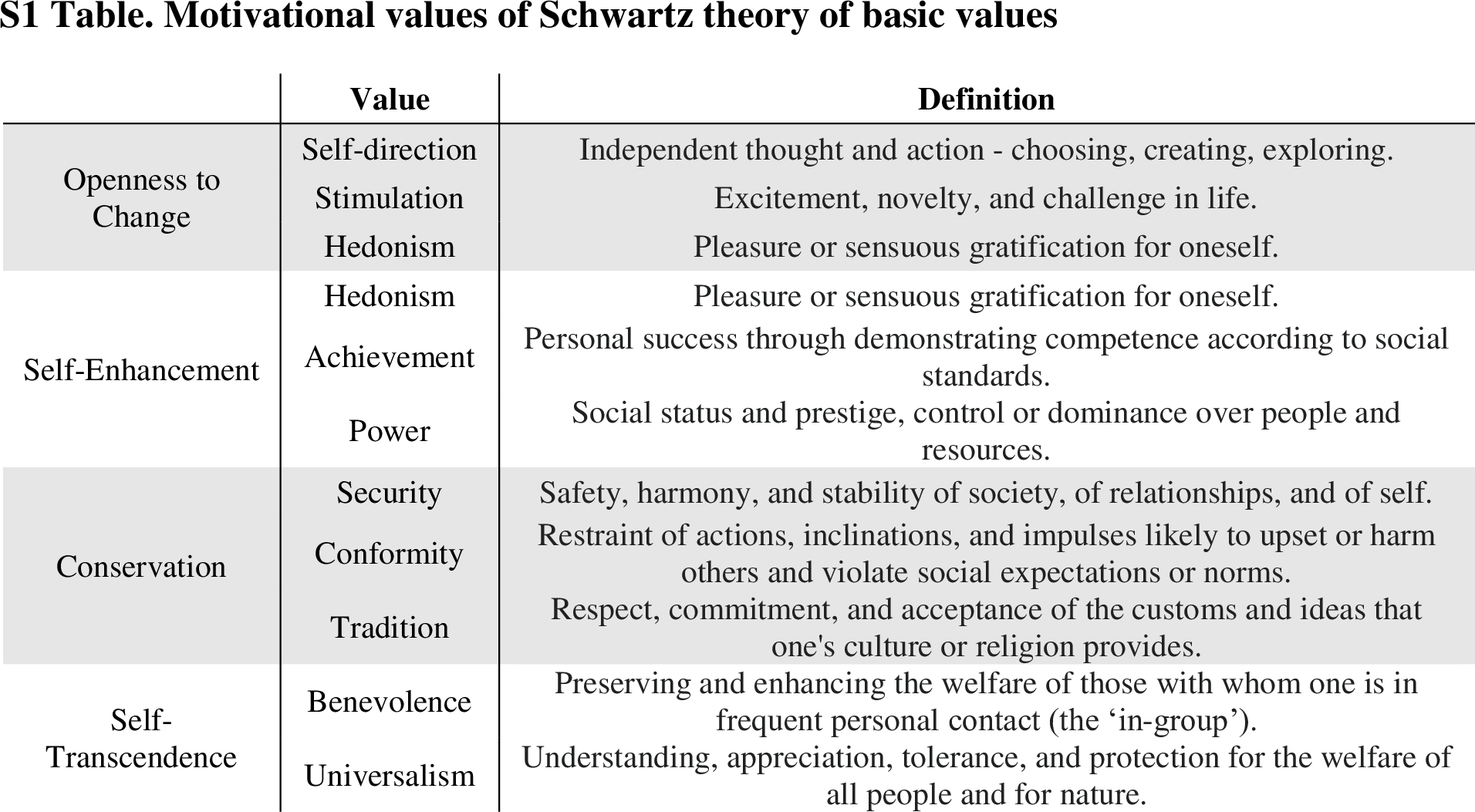

Supplement: S1 Table — (TIF) [file pone.0294531.s001.tif]

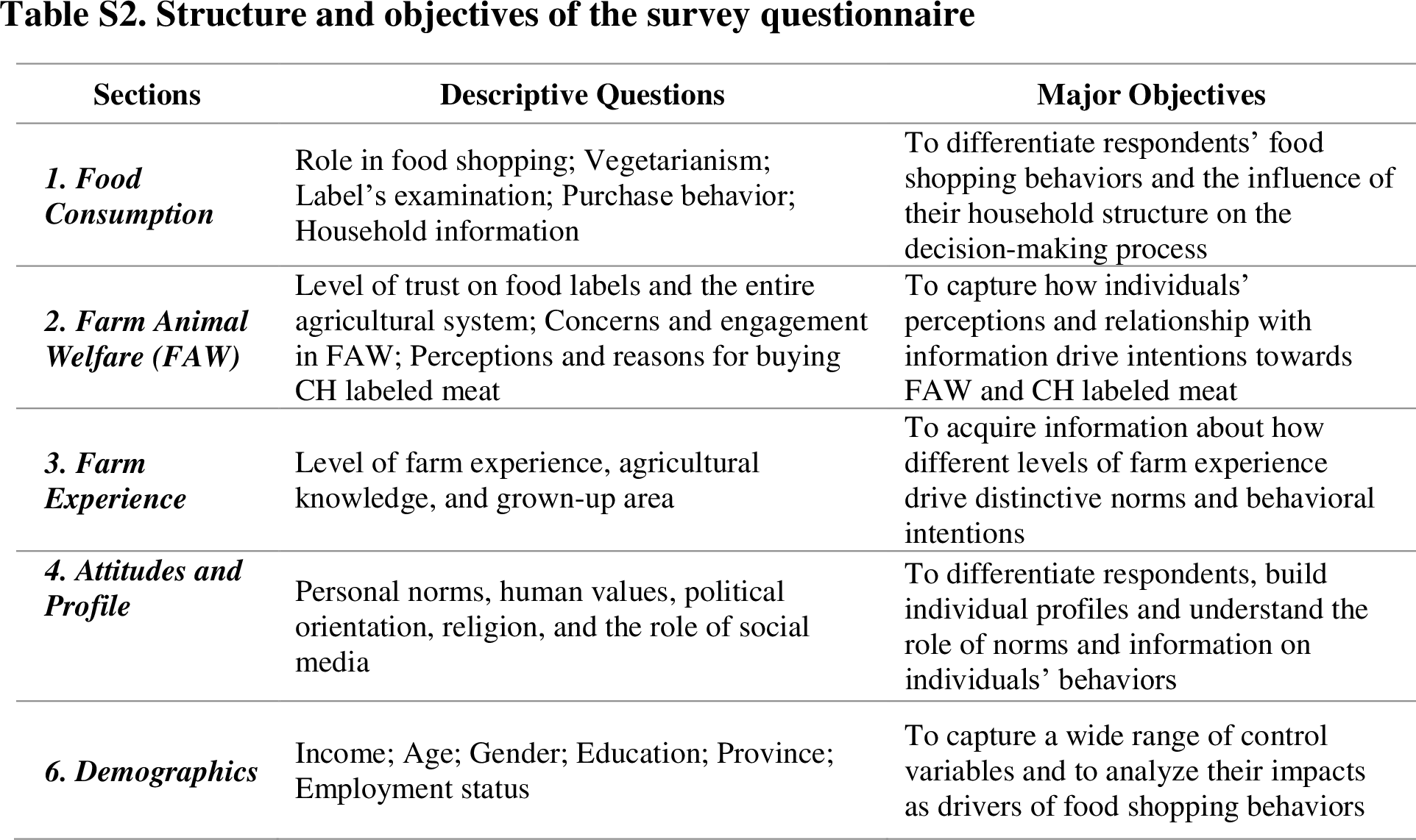

Supplement: S2 Table — (TIF) [file pone.0294531.s002.tif]

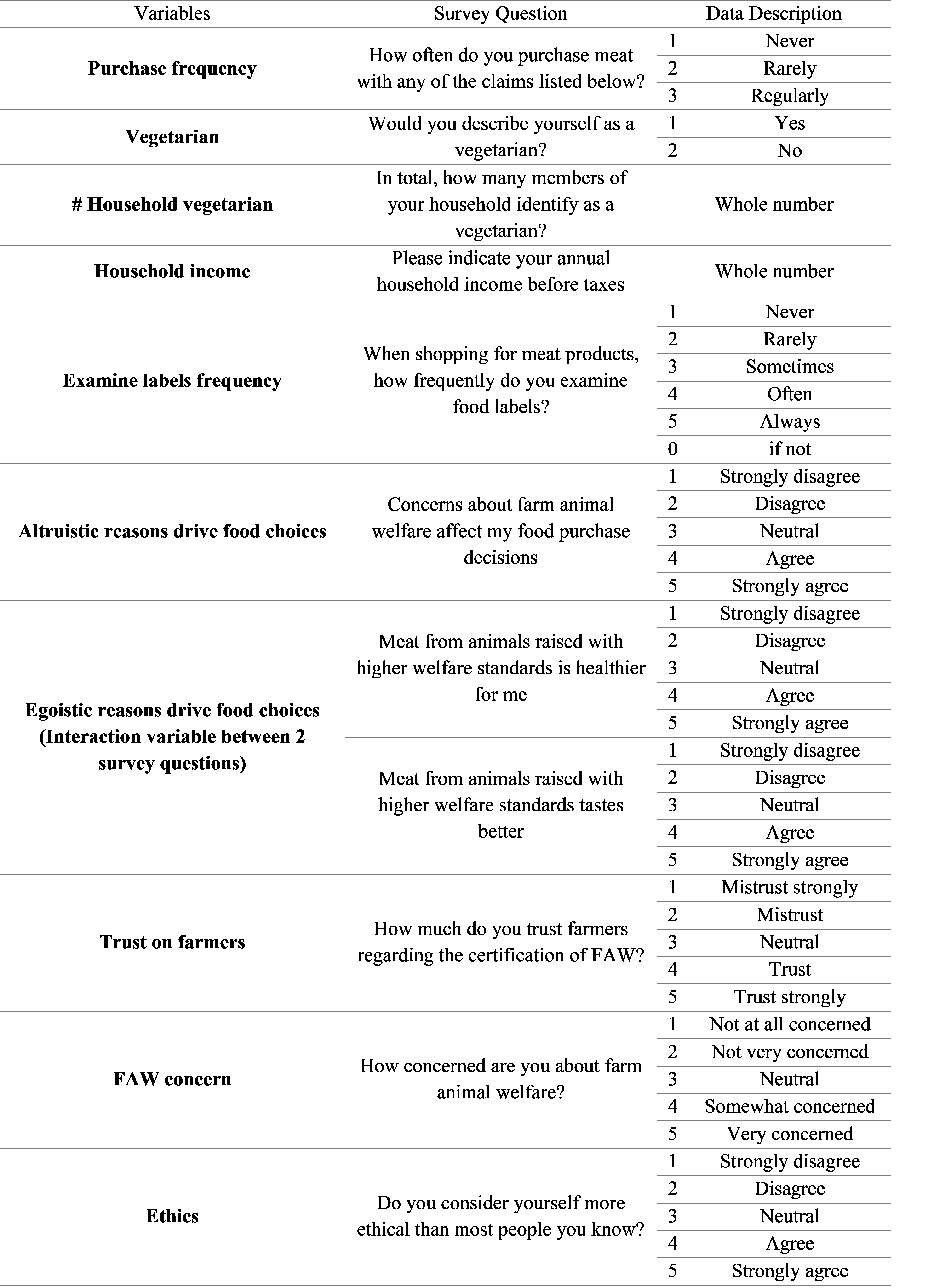

Supplement: S3 Table — (TIF) [file pone.0294531.s003.tif]

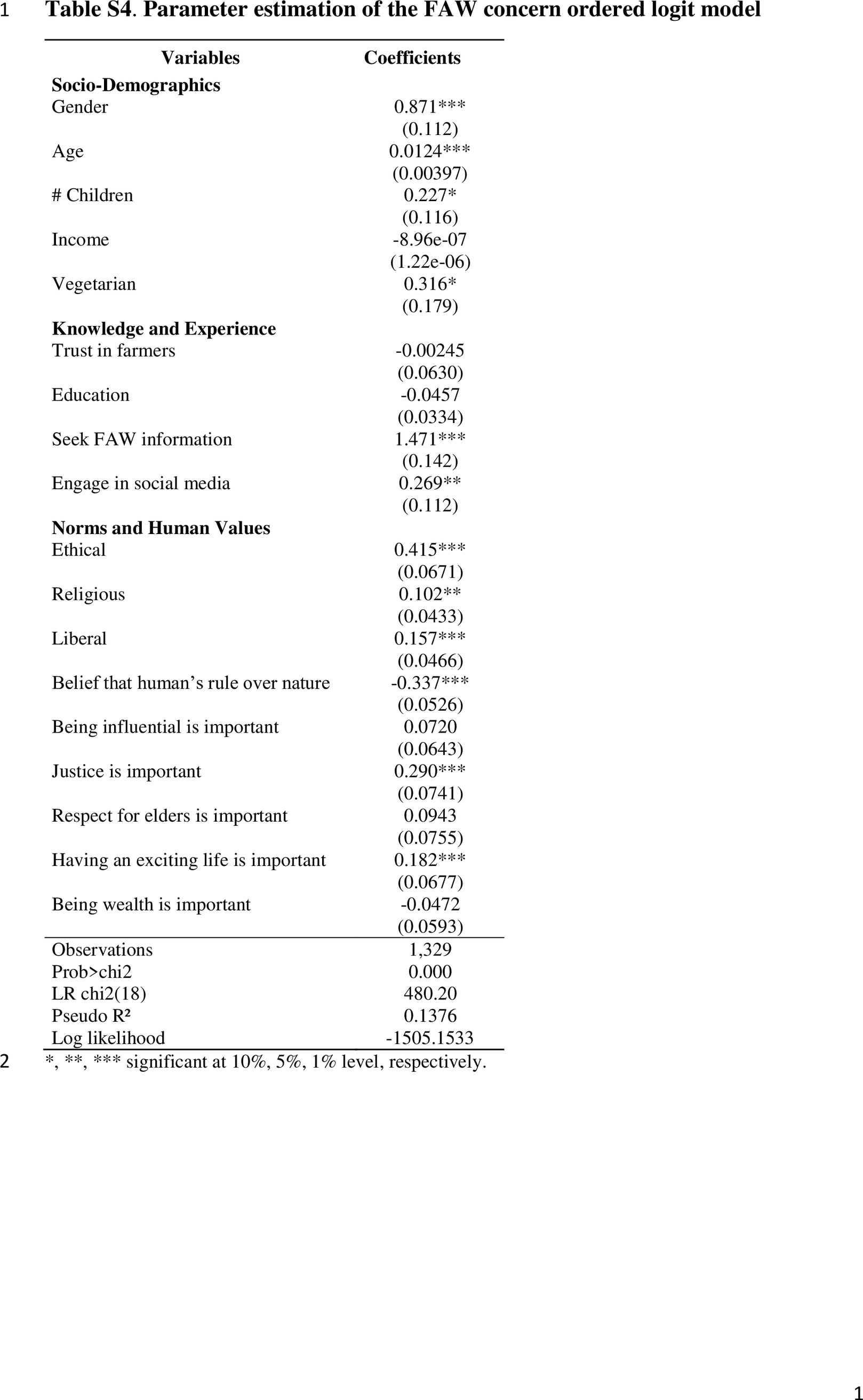

Supplement: S4 Table — (TIF) [file pone.0294531.s004.tif]
